# Association of SARS-CoV-2 Seropositive Antibody Test With Risk of Future Infection

**DOI:** 10.1001/jamainternmed.2021.0366

**Published:** 2021-02-24

**Authors:** Raymond A. Harvey, Jeremy A. Rassen, Carly A. Kabelac, Wendy Turenne, Sandy Leonard, Reyna Klesh, William A. Meyer, Harvey W. Kaufman, Steve Anderson, Oren Cohen, Valentina I. Petkov, Kathy A. Cronin, Alison L. Van Dyke, Douglas R. Lowy, Norman E. Sharpless, Lynne T. Penberthy

**Affiliations:** 1 Aetion, Inc, New York, New York; 2 HealthVerity, Philadelphia, Pennsylvania; 3 Quest Diagnostics, Secaucus, New Jersey; 4 LabCorp, Burlington, North Carolina; 5 National Cancer Institute, National Institutes of Health, Bethesda, Maryland

## Abstract

**Importance:**

Understanding the effect of serum antibodies to severe acute respiratory syndrome coronavirus 2 (SARS-CoV-2) on susceptibility to infection is important for identifying at-risk populations and could have implications for vaccine deployment.

**Objective:**

The study purpose was to evaluate evidence of SARS-CoV-2 infection based on diagnostic nucleic acid amplification test (NAAT) among patients with positive vs negative test results for antibodies in an observational descriptive cohort study of clinical laboratory and linked claims data.

**Design, Setting, and Participants:**

The study created cohorts from a deidentified data set composed of commercial laboratory tests, medical and pharmacy claims, electronic health records, and hospital chargemaster data. Patients were categorized as antibody-positive or antibody-negative according to their first SARS-CoV-2 antibody test in the database.

**Main Outcomes and Measures:**

Primary end points were post-index diagnostic NAAT results, with infection defined as a positive diagnostic test post-index, measured in 30-day intervals (0-30, 31-60, 61-90, >90 days). Additional measures included demographic, geographic, and clinical characteristics at the time of the index antibody test, including recorded signs and symptoms or prior evidence of coronavirus 2019 (COVID) diagnoses or positive NAAT results and recorded comorbidities.

**Results:**

The cohort included 3 257 478 unique patients with an index antibody test; 56% were female with a median (SD) age of 48 (20) years. Of these, 2 876 773 (88.3%) had a negative index antibody result, and 378 606 (11.6%) had a positive index antibody result. Patients with a negative antibody test result were older than those with a positive result (mean age 48 vs 44 years). Of index-positive patients, 18.4% converted to seronegative over the follow-up period. During the follow-up periods, the ratio (95% CI) of positive NAAT results among individuals who had a positive antibody test at index vs those with a negative antibody test at index was 2.85 (95% CI, 2.73-2.97) at 0 to 30 days, 0.67 (95% CI, 0.6-0.74) at 31 to 60 days, 0.29 (95% CI, 0.24-0.35) at 61 to 90 days, and 0.10 (95% CI, 0.05-0.19) at more than 90 days.

**Conclusions and Relevance:**

In this cohort study, patients with positive antibody test results were initially more likely to have positive NAAT results, consistent with prolonged RNA shedding, but became markedly less likely to have positive NAAT results over time, suggesting that seropositivity is associated with protection from infection. The duration of protection is unknown, and protection may wane over time.

## Key Points

**Question:**

Can observational clinical data from commercial laboratories be used to evaluate the comparative risk of severe acute respiratory syndrome coronavirus 2 (SARS-CoV-2) infection for individuals who are antibody positive vs those who are antibody negative?

**Finding:**

In this cohort study of more than 3.2 million US patients with a SARS-CoV-2 antibody test, 0.3% of those indexed with positive test results had evidence of a positive nucleic acid amplification test beyond 90 days after index, compared with 3.0% indexed with negative antibody test results.

**Meaning:**

Individuals who are seropositive for SARS-CoV-2 based on commercial assays may be at decreased future risk of SARS-CoV-2 infection.

## Teaser

This cohort study evaluates evidence of SARS-CoV-2 infection based on diagnostic nucleic acid amplification test among patients who tested positive versus negative for antibodies.

## Introduction

Since the emergence of severe acute respiratory syndrome coronavirus 2 (SARS-CoV-2) in late 2019, limited research has shown that the majority of patients who clear their infections develop serum antibodies against the virus that last for at least several months^1,2,3,4,5,6^ but may decline over time.^7^ Although it has been speculated that the development of antibodies may be associated with a decreased risk of reinfection, the evidence for this hypothesis is limited and often anecdotal.^8,9^ Furthermore, documented reports of reinfection in patients with SARS-CoV-2 antibodies have raised the possibility that seropositivity might be associated with limited protection against different viral strains.^10,11,12,13,14^ Individuals infected with SARS-CoV-2 may also shed viral RNA without producing live virus for 12 weeks or more after resolution of symptoms,^15,16,17,18,19,20^ making it challenging to distinguish reinfection from prolonged RNA shedding. As the coronavirus disease 2019 (COVID-19) pandemic continues, understanding the role of serostatus on the potential for infection is critical, as it may drive choices of personal behavior and expectations about herd immunity. It might also help inform the challenging policy decisions surrounding the prioritization of vaccine supplies.

Commercially available antibody assays, with their high sensitivity and low false-positive rates,^21,22,23^ serve as a useful marker of prior SARS-CoV-2 infection, but to date, their ability to predict the risk of future infection is unknown. Given the critical lack of data in this area, the US Centers for Disease Control currently recommend that individual serology results not be used for any decision-making regarding personal behavior (such as return to work, use of personal protective equipment, and social distancing). These gaps highlight the clear need for generalizable data that can elucidate the effect of seropositivity on risk of future infection. This type of observational data, often referred to as real-world data,^24,25^ represents an opportunity as they are available longitudinally at the individual level and make it possible to study the experiences of a seropositive population with COVID-19 in near-real time, while maximizing sample size and observability over time.

In this article, we employ an approach leveraging a large set of clinical laboratory data linked to other clinical information such as claims and chargemaster information to investigate the relationship between SARS-CoV-2 antibody status and subsequent nucleic acid amplification test (NAAT) results, in an effort to understand how serostatus may predict risk of reinfection.

## Methods

In this retrospective observational descriptive cohort study, we used deidentified individual-level laboratory testing data provided by HealthVerity (Philadelphia, PA), a for-profit data aggregator that provides access to linked data from 70 different commercial health data sources. Data available for this study included results from several national and regional clinical commercial laboratories, representing more than 50% of commercial antibody and diagnostic testing in the US (see eFigure 1 and eFigure 2 in the Supplement). These longitudinally linked commercial laboratory data were the primary data sources for this study’s analyses. In addition, longitudinal data on each individual were captured from open and closed medical and pharmacy claims, electronic health records, and hospital billing records from multiple vendors (see details in eAppendix in the Supplement). These data were used to assess the availability of data to characterize patient-level comorbid conditions and other risk factors that might affect infection risk and outcome. The data derived from laboratories, medical record systems, and insurance claims cover the US but may under-sample the Midwest region. To create the consolidated, deidentified data set with longitudinal patient views, all data partners used the HealthVerity technology within their system to create a unique, secure, encrypted, and nonidentifiable patient token from identifiable information. This token was then employed as a consistent linkage key across data sets, and enabled follow-up of patients who, for example, used multiple laboratory providers. No protected health information or personal identifying information left the data owner’s possession, and all research data were certified by expert determination to be compliant with the Health Insurance Portability and Accountability Act rules. As part of this process, race and ethnicity were removed from the files. To maintain nonidentifiability of patients, race and ethnicity information was not available in the research data set.

Study reporting follows the Strengthening the Reporting of Observational Studies in Epidemiology (STROBE) reporting guideline for observational studies.^26^ The study was approved under exemption by the New England Institutional Review Board (#1-9757-1).

The antibody testing performed by commercial laboratories includes a limited set of high-throughput antibody tests with validation against a known standard providing between 98% and 100% agreement with both known antibody-positive and antibody-negative specimens, with a 95% CI of 99% to 100% agreement. An evaluation of the US Food and Drug Administration emergency use authorization documents shows that the composite negative validation data demonstrate a 95% confidence interval range of 99% to 100%.^22,23^ Tests performed in these commercial laboratories are those specific for immunoglobulin (Ig) G, IgA, or IgM, as well as those that detect multiple immunoglobulin types, although most tests performed during the study period were IgG (>91%).

**Figure 1.**
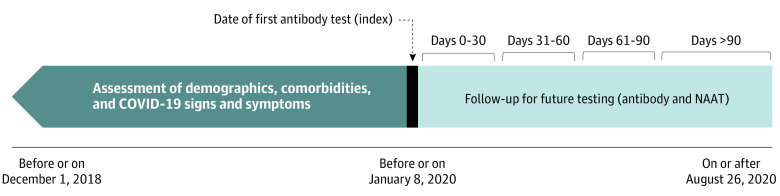
Diagram of Study Design This figure shows the key elements of the study design. The study index date for each patient was the day of the patient’s first observed severe acute respiratory syndrome coronavirus 2 (SARS-CoV-2) antibody test on or after January 8, 2020. Follow-up occurred in 30-day increments after the index date. COVID-19 indicates coronavirus disease 2019; NAAT indicates nucleic acid amplification test.

We examined records from December 1, 2018, through August 26, 2020, and identified individuals with a recorded SARS-CoV-2 antibody test on or after January 2020. Each patient entered the cohort on the day of their first recorded antibody test, which was defined as the index date (see Figure 1). Individuals who had more than 1 antibody test with discordant results on the index day were excluded. Using our linked longitudinal data set, we assessed demographic and geographic characteristics at index, as well as evidence of prior SARS-CoV-2 infection and key associated clinical characteristics and comorbidities. These characteristics were measured as recorded in the EHR, administrative claims, and hospital records.

**Figure 2.**
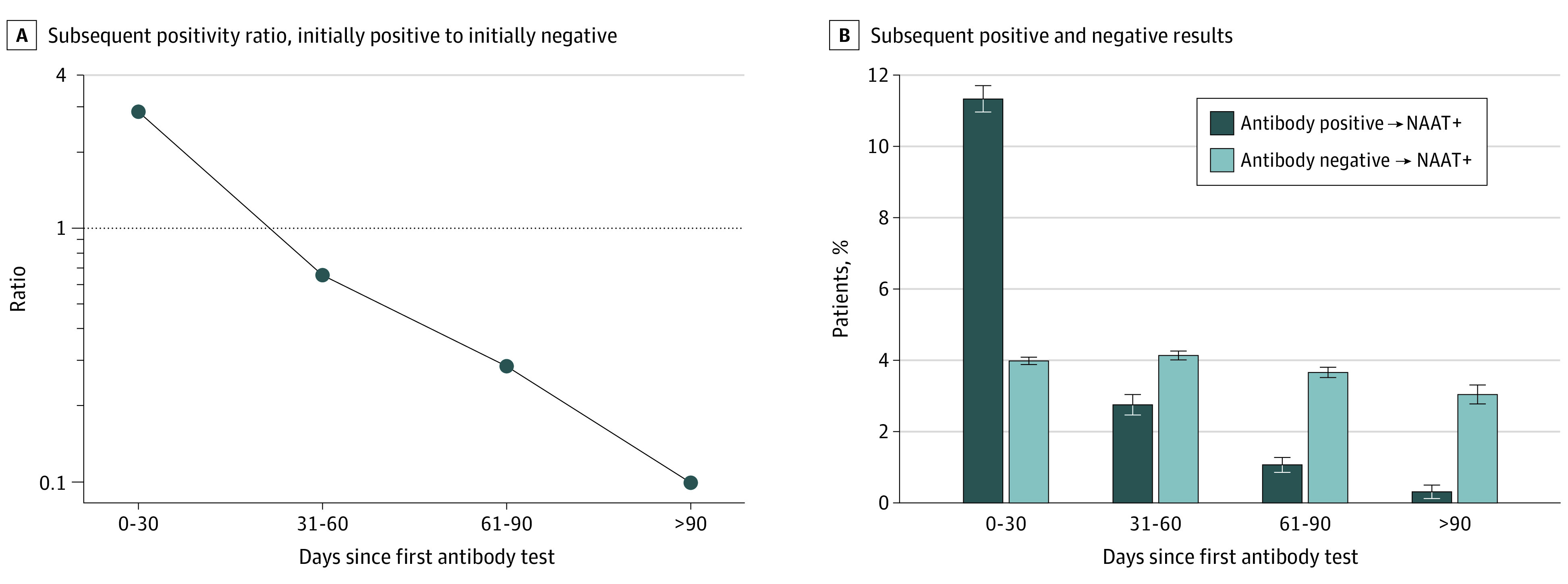
Subsequent Diagnostic Nucleic Acid Amplification Test (NAAT) Results at 30-Day Intervals This figure shows the results of diagnostic NAAT after initial antibody testing. A, The line shows the ratio of positive diagnostic tests among those who initially tested positive for antibodies vs those who initially tested negative. B, Over each time period, the dark blue bars show the percent of patients who tested positive for the diagnostic test among those who initially tested positive for antibodies with corresponding confidence intervals. The light blue bars show the percent of patients who tested positive for the diagnostic test among those who initially tested negative for antibodies with corresponding confidence intervals.

We characterized patients’ initial antibody test results as positive, negative, or inconclusive, and created 3 associated groups. We then followed patients to the end of available data (August 26, 2020) to identify further antibody testing and/or NAAT diagnostic testing, looking in 30-day intervals (0-30, 31-60, 61-90, >90 days). Within each interval and for each of the 3 index antibody groups, we assessed both the frequency of subsequent antibody or NAAT diagnostic testing and the test results. An individual was characterized as testing positive for an antibody or NAAT during a time period if they had at least 1 positive test during that period. Patients were counted uniquely within each time period and could have been included in multiple time periods. All analyses were done on the Aetion Evidence Platform (Aetion, Inc, New York, NY), version R4.11. Confidence intervals around the ratio of proportions were estimated using the natural logarithm method for the rate ratios presented in Figure 2.

## Results

A total of 3 257 478 unique patients with an index antibody test were identified after excluding 132 patients with discordant antibody tests on the index day. Of these, 2 876 773 (88.3%) had a negative index antibody result (seronegatives), 378 606 (11.6%) had a positive index antibody result (seropositives), and 2099 (0.1%) had an inconclusive index antibody result (sero-uncertain) (Table). As the sero-uncertain group was a small fraction of the study population, further reported results focus only on the seropositive and seronegative groups. Approximately 55% in each group were female. The index seronegative group was somewhat older than the index seropositive group (mean [SD] of 48 [17.6] vs 44 [18.1] years). A higher proportion of index seropositive individuals resided in the Northeast United States, with fewer in the rest of the country (eFigure 1 in the Supplement). The seropositive and seronegative groups each had a median of 396 days of observable person-time prior to the index date. Over that time, most COVID-19 signs and symptoms were similar between the seropositive and seronegative groups, although the seropositive groups had higher proportions of recorded fever (6.3% among seropositives vs 3.5% among seronegatives), acute respiratory failure (1.2% vs 0.4%), and viral infection (4.3% vs 2.0%). Other comorbidities were largely comparable between the seropositive and seronegative groups, with the exceptions of obesity (19.5% vs 16.8%) and vitamin D deficiency (14.5% vs 12.3%), which were slightly higher among individuals who were seropositive than seronegative (Table). As expected, evidence of prior COVID-19 diagnosis varied across the 3 groups. Evidence of prior disease based on laboratory, claims, and/or chargemaster diagnostic codes was 0.7% for the seronegative group, 18.4% for the seropositive group, and 6.7% for the sero-uncertain group. These results indicate that seropositive individuals were more likely to have had symptoms of and/or a diagnosis of COVID-19 than seronegative individuals, although the majority of subjects in both groups lacked evidence of prior infection in the observable data.

**Table.** Baseline and Preindex Characteristics

Characteristic	Index (first) antibody test result, total (n = 3 257 478)		
	Negative result	Positive result	Inconclusive result
No. (%)	2 876 773 (88.3)	378 606 (11.6)	2099 (0.1)
Demographic characteristics			
Age, y			
Mean (SD)	47.66 (17.63)	44.34 (18.09)	49.45 (19.22)
Median (IQR)	48.00 (34-61)	45.00 (30-58)	50.00 (35-64)
Sex, No. (%)			
Male	1 219 912 (43.2)	171 240 (45.8)	922 (44.6)
Female	1 599 898 (56.7)	202 157 (54.1)	1143 (55.3)
Geographic region, No. (%)			
Northeast	1 008 720 (35.8)	230 513 (61.7)	305 (14.8)
Midwest	239 837 (8.5)	16 735 (4.5)	56 (2.7)
South	786 551 (27.9)	51 648 (13.8)	403 (19.5)
West	514 441 (18.2)	26 706 (7.1)	1066 (51.6)
Index antibody test type, No. (%)			
Antibody	237 035 (8.2)	49 414 (13.1)	3 (0.1)
Antibody IgA	1782 (0.1)	50 (0)	19 (0.9)
Antibody IgG	2 625 428 (91.3)	328 506 (86.8)	1648 (78.5)
Antibody IgM	12 528 (0.4)	636 (0.2)	429 (20.4)
Patient comorbidities			
Chronic conditions, No. (%)^a^			
Hypertension	430 516 (24.2)	52 700 (24.7)	429 (30.8)
Ischemic heart disease	96 920 (5.4)	10 423 (4.9)	137 (9.8)
Coronary heart disease	80 730 (4.5)	8333 (3.9)	118 (8.5)
Metabolic syndrome	42 549 (2.4)	6244 (2.9)	41 (2.9)
Vitamin D deficiency	219 142 (12.3)	30 930 (14.5)	145 (10.4)
Obesity	311 393 (16.8)	42 890 (19.5)	301 (20.7)
Preindex COVID-19 diagnosis^a^			
Patients with preindex diagnosis, No. (%)	11 305 (0.4)	23 824 (6.8)	52 (2.6)
Median days to most recent preindex diagnosis (IQR)	1.00 (1-19)	18.00 (2-38)	9.50 (1-24)

Abbreviations: COVID-19, coronavirus disease 2019; Ig, immunoglobulin; IQR, interquartile range.

^a^ Based on medical claims and chargemaster data.

**Figure 3.**
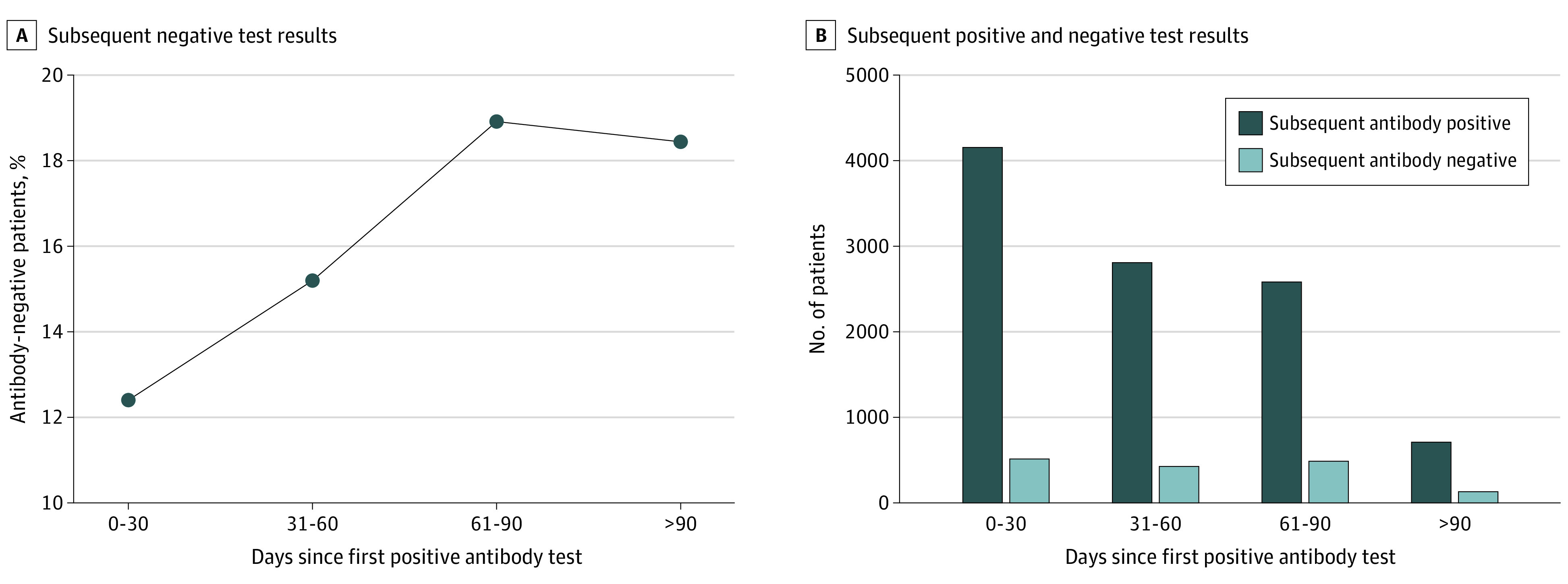
Subsequent Antibody Testing Among Index Antibody-Positive Patients Over Time This figure shows the results of subsequent antibody tests among the group of patients with an initial positive antibody test (n = 378 606). A, The line shows the percentage of patients who subsequently tested negative in each time period. B, Over the 4 time periods, light blue bars represent those who subsequently tested negative for antibodies, while dark blue bars show those who subsequently tested positive.

The linked data permitted individual longitudinal follow-up for a median of 47 days (interquartile range [IQR], 8 to 88 days) for the seronegative group and a median of 54 days (IQR, 17 to 92 days) for the seropositive group. Over the available follow-up time, we examined the duration of seropositivity in the index positive cohort. Among the 378 606 patients with a positive antibody test at index, 9895 (2.6%) had at least one subsequent antibody test during follow-up. For the index seropositive patients who were retested, 12.4% tested negative when retested within 0 to 30 days, increasing to 18.4% testing seronegative when the subsequent antibody test occurred more than 90 days after the index antibody test (Figure 3). These findings are consistent with prior studies suggesting that antibody levels wane in a modest fraction of individuals over a period of months after initial detection.^1,2,3^

We next considered the relationship between index serostatus and future NAAT testing patterns. Among the seropositive patients, 41 587 (11.0%) had 1 or more NAAT during follow-up, while among seronegative patients, 273 735 (9.5%) did so. Patients may have had multiple NAATs during follow-up; seropositive patients had a mean of 3.3 NAATs over the follow-up period, while seronegative patients had 2.3 tests on average. Sero-uncertain patients were tested less frequently, with 1.5 tests per patient performed on average.

Among patients with a positive index antibody result, 3226 (11.3%) had a positive diagnostic NAAT during follow-up that occurred within 30 days of index, decreasing consistently to 2.7% from 31 to 60 days, 1.1% from 61 to 90 days, and 0.3% at more than 90 days (Figure 2). For the seronegative patients, 5638 (3.9%) showed a positive NAAT result within 30 days. That proportion remained relatively consistent at approximately 3.0% over all subsequent periods of observation, including after 90 days (Figure 3). The ratio of positive NAAT results among patients who had a positive antibody test at index vs those with a negative antibody test at index declined from 2.85 (95% CI, 2.73-2.97) at 0 to 30 days; to 0.67 (95% CI, 0.6-0.74) at 31 to 60 days; to 0.29 (95% CI, 0.24-0.35) at 60 to 90 days; and to 0.10 (95% CI, 0.05-0.19) at more than 90 days.

## Discussion

Early in the observation period, particularly in the first 30 days, positive NAAT results among seropositive patients are likely attributable to prolonged shedding of viral RNA, which is expected to decrease through the following weeks. The increased rate of NAAT result positivity observed within the first 30 days of a positive antibody test is consistent with persistent shedding of viral RNA.^15,16,17,18,19,20^ Beyond 90 days, the vast majority of viral shedding is expected to have ceased, so positive NAAT results seen at a later interval from the index antibody test may represent new infections. False positives are expected to be rare given the high specificity of NAAT, and they are thought to generally reflect technical errors or reagent contamination (the latter is less likely due to internal controls).^27,28,29^ Under the assumptions that positive diagnostic tests among seronegative patients represent infections and that positive diagnostic tests among patients who first tested seropositive more than 90 days prior also represent infections, we observed 2 notable results. First, the relatively steady approximately 3.0% proportion of positive NAATs among index seronegative patients suggests a stable background infection rate over the study period.

Second, while our study was not appropriate for estimating a relative risk, the ratio of positive NAAT results among index seropositive individuals compared with index seronegative individuals was substantially lower—an approximately 10-fold decrease—suggesting a protective effect of antibodies. While some patients may have ongoing viral RNA shedding for weeks after infection, the sharp decline in NAAT-positive results over time in the antibody-positive cohort vs antibody-negative cohort suggests that seropositive individuals are at decreased risk for future SARS-CoV-2 infection. As the pandemic infection rates varied both over time and by geographic area, we performed a preliminary stratified analysis that evaluated the risk of subsequent infection by geographic region in the United States. Although the numbers were small for some regions, the results showed a consistent decline in the ratio of NAAT positivity among seropositive vs seronegative patients in all regions over the 4 study intervals, similar to the overall analysis. This consistency supports the same level of reduction in future risk and is unlikely to be attributable to pandemic patterns of testing and/or spread (data shown in eTable in the Supplement). The degree of protection (10-fold) associated with seropositivity appears to be comparable to that observed in the initial reports of the efficacy of mRNA vaccines in large clinical trials.^30,31,32^ Of course, protection induced by a safe vaccine is clearly preferable, as the population-wide risk of a serious outcome from an authorized or approved vaccine is expected to be orders of magnitude lower than that from natural infection. Additionally, this study corroborates the findings reported by Lumley et al^33^; however, the cohort in this study is larger and more generalizable to the general population as it extends beyond health care workers.

### Limitations

Given the observational nature of the study, it is possible that antibody test results affected individual behavior, potentially confounding the results. We do not, however, think that behavior differences are likely to explain the observed protection. For example, if individuals with evidence of prior infection (seropositive individuals) were more likely to believe they possessed immunity to SARS-CoV-2, then they would be expected to engage in social behavior that placed them at greater, not less, risk for infection. Likewise, it is possible that seropositive individuals might be less likely to seek evaluation for subsequent symptoms of COVID-19, but, in fact, we observed that antibody-positive individuals were more likely to have follow-up NAAT than antibody-negative individuals (3.3 vs 2.3 subsequent tests).

We do not have insight into the clinical characteristics of the seropositive individuals who appeared to develop new infections after the index time point, nor could we specifically assess the clinical course of these possible infections compared with infections among the seronegative group in this study. However, some of the individuals who had NAAT-positive results more than 60 days after an index seropositive test may represent true infections, as reinfection has been described in a small number of cases.^8,9,10,11^ Therefore, on a population-wide basis, protection against reinfection is likely relative rather than complete. Factors that influence reinfection risk—such as varying viral strains, patients’ immune status, or other patient-level characteristics—should be evaluated in subsequent studies that include follow-up beyond 90 days. There is limited but consistent evidence from two SARS-CoV-2 outbreaks suggesting that seropositivity is associated with protection from infection. In an outbreak on a fishing vessel, an attack rate of 85% was observed among the 122 individuals. Only 3 individuals aboard were known to have serum neutralizing antibodies prior to the outbreak, and none of them became infected.^9^ In another outbreak, at a children’s summer camp, 116 out of 156 total (76%) campers, counselors, and staff became infected, but all 24 of the individuals who were seropositive when the camp began tested negative for infection soon after the epidemic had subsided.^8^ The current findings extend those anecdotal series onto a much larger sample size based on commercially available assays used in settings outside clinical trials.

While there are acknowledged limitations to observational clinical data, these data do provide a means to complement and supplement data from clinical trials in order to formulate hypotheses and provide information on patients or clinical scenarios that are not well represented in trials.^34,35,36,37^ It is particularly well suited to situations such as an emerging pandemic, where urgent questions require rapid, near real-time answers.

To be clear, however, this analysis based on nonrandomized observational data from commercial laboratories and claims has significant limitations compared with a classical prospective seroprotection trial. First, it is not known whether the rate of SARS-CoV-2 exposure or pattern of longitudinal follow-up were comparable between the 2 groups. It is also not known whether the positive NAAT results in either group were associated with clinical signs of infection. Perhaps most importantly, it is not known how long any protective effect of serostatus may last beyond the studied days. These questions remain to be addressed by further research. That research can also shed light on whether a seropositive individual who subsequently becomes seronegative may have reduced protection and the degree to which protection associated with seropositivity may be mediated by antibodies vs other forms (eg, T-cell based) of immunity.^6^

## Conclusions

In this cohort study, deidentified data from commercial laboratories suggest that the presence of antibodies to SARS-CoV-2 is associated with a reduced risk of having a subsequent positive NAAT results, which may be a proxy representing a new infection or may represent continued viral shedding depending on the context and timing. While this risk reduction was not seen in the first 30 days after an initial antibody test, it became pronounced after 30 days and progressively strengthened through the 90-day observation period and beyond.

## Notes

Supplement. eFigure 1. Rates of index seronegativity by US state eFigure 2. Rates of index seropositivity by US state eAppendix. HealthVerity Data eTable. Ratio of NAAT positivity among index antibody positive and negative by geographic region and observation period (95% CI)
