# Supplementary material for: Association of SARS-CoV-2 Seropositive Antibody Test With Risk of Future Infection
Source: JAMA Intern Med. 2021 Feb 24;181(5):672–9. doi: 10.1001/jamainternmed.2021.0366 (PMC7905701; doi:10.1001/jamainternmed.2021.0366)
Supplement: Supplement. — eFigure 1. Rates of index seronegativity by US state eFigure 2. Rates of index seropositivity by US state eAppendix. HealthVerity Data eTable. Ratio of NAAT positivity among index antibody positive and negative by geographic region and observation period (95% CI) [file jamainternmed-e210366-s001.pdf]

# Supplemental Online Content

Harvey RA, Rassen JA, Kabelac CA, et al. Association of SARS-CoV-2 seropositive antibody test with risk of future infection. *JAMA Intern Med*. Published online February 24, 2021. doi:10.1001/jamainternmed.2021.0366

**eFigure 1.** Rates of index seronegativity by US state
**eFigure 2.** Rates of index seropositivity by US state
**eAppendix.** HealthVerity Data
**eTable.** Ratio of NAAT positivity among index antibody positive and negative by geographic region and observation period (95% CI)

This supplemental material has been provided by the authors to give readers additional information about their work.

© 2021 Harvey RA et al. *JAMA Internal Medicine.*

**eFigure 1. Rates of index seronegativity by US state**

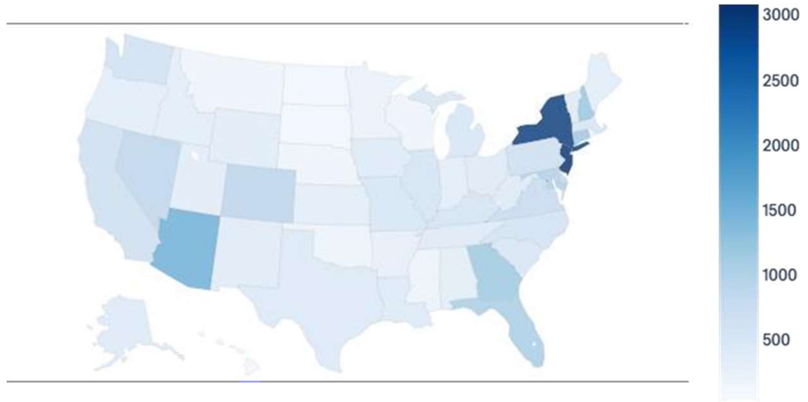


This figure shows the frequency of index seronegativity (blue) per 100,000 residents of each US state.

© 2021 Harvey RA et al. *JAMA Internal Medicine.*

**eFigure 2. Rates of index seropositivity by US state**

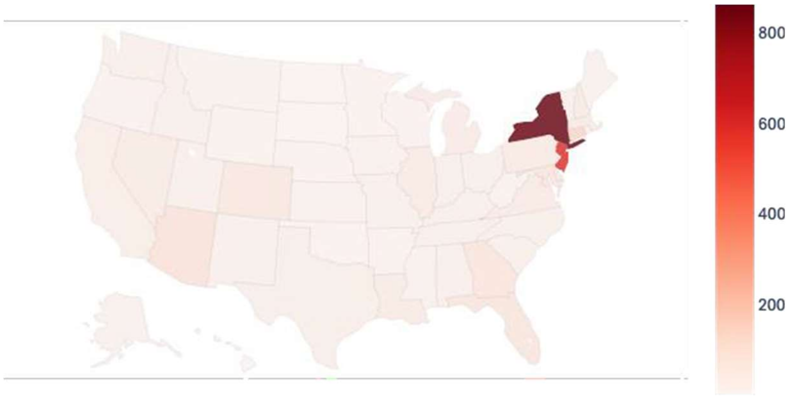


This figure shows the frequency of index seropositivity (red) per 100,000 residents of each US state.

© 2021 Harvey RA et al. *JAMA Internal Medicine.*

**eAppendix. HealthVerity Data**

HealthVerity data contain near real-time medical claims and outpatient pharmacy transactions, including drugs, diagnoses, procedures and selected lab results (incl. COVID-19 testing). These data are drawn from a variety of US sources which include Veradigm and over 70 other HealthVerity data partners. Data elements include provider-submitted claims, adjudicated insurance claims, pharmacy billing managers claims submissions, and US laboratory chain test orders and selected results. They update in near real time, with minimal lag between time of claim submission and time of inclusion in the database. 12+ months of historical data is available for many patients. Hospitalizations are included in the data at a summary level. Further, near real-time hospital chargemaster data, consisting of data from internal hospital billing systems, and capturing all billable drugs, procedures, and medical services provided to a patient in an in-hospital setting, are available (without history) for select patients. Data are augmented for select patients with detailed clinical data from electronic health records. As information about comorbidities and other risk factors is drawn from these data sources, recorded values may underrepresent conditions that are not well-reported in insurance claims or require detailed historical hospital information. For reported baseline use of medications, drugs dispensed by a pharmacy are generally very well captured, though OTC medication use may not be.

All data include key factors such as patient age, gender, and 3-digit zip level and may include an identifier for the treating provider. Data are de-identified and were certified HIPAA-compliant by expert determination.

Death information is generally available with minimal lag for patients who die in a hospital setting, and for other settings may also be linked from other data sources (with additional lag time).Each dataset is provided by linking records on a unique patient identifier created by HealthVerity. The linkage of patients has high accuracy: 99.7% of linkages made are made correctly (0.3% false positives), and 96% of possible linkages are made (4% false negative). All linkage is done according to HIPAA regulations. With real-time assembly of data requiring the use of multiple sources, this approach appropriately balances timeliness with fidelity of linkage.

© 2021 Harvey RA et al. *JAMA Internal Medicine.*

**eTable**

| Ratio of NAAT Positivity Among Index Antibody Positive and Negative By Geographic Region and Observation Period (95% CI) | | | | |
|---|---|---|---|---|
| | *Follow-up Period* | | | |
| *Region* | 0-30 days | 31 -60 days | 61-90 days | 90+ days |
| Overall | 2.85 (2.73 - 2.97) | 0.67 (0.6 - 0.74) | 0.29 (0.24 - 0.35) | 0.1 (0.05 - 0.19) |
| Northeast | 5.39 (5.01 - 5.80) | 1.86 (1.60 - 2.15) | 0.75 (0.58 - 0.98) | 0.22 (0.09 - 0.49) |
| Midwest | 3.89 (3.15 - 4.79) | 1.13 (0.67 - 1.92) | 0.63 (0.24 - 1.70) | 0 (0.0 - 0.0) |
| South | 2.14 (1.98 - 2.31) | 0.53 (0.39 - 0.72) | 0.36 (0.22 - 0.58) | 0.21 (0.05 - 0.84) |
| West | 3.62 (3.23 - 4.07) | 0.69 (0.44 - 1.07) | 0.46 (0.21 - 1.02) | 0 (0.0 - 0.0) |
| Unknown | 3.40 (2.93 - 3.95) | 0.62 (0.38 - 1.01) | 0.26 (0.11 - 0.58) | 0.31 (0.07 - 1.27) |

Abbreviation: NAAT indicates nucleic acid amplification test. This table provides a preliminary analysis of the ratio of NAAT positivity in index antibody positive versus index antibody negative patients by geography and time interval. Results are consistent with overall analysis.

© 2021 Harvey RA et al. *JAMA Internal Medicine.*
